# Acute stress reduces out-group related safety signaling during fear reinstatement in women

**DOI:** 10.1038/s41598-020-58977-6

**Published:** 2020-02-07

**Authors:** Christian Josef Merz, Annika Eichholtz, Oliver Tobias Wolf

**Affiliations:** Ruhr University Bochum, Faculty of Psychology, Institute of Cognitive Neuroscience Department of Cognitive Psychology, Universitätsstr. 150, 44780 Bochum, Germany

**Keywords:** Fear conditioning, Stress and resilience, Human behaviour

## Abstract

When using in-group and out-group faces as conditional stimuli (CS) in fear conditioning designs, extinction learning is selectively impaired for out-group faces. Additionally, stress seems to inhibit extinction retrieval leading to a higher return of fear, which might be especially the case for out-group faces. To test this hypothesis, 51 healthy women underwent fear acquisition training, consisting of repeated presentations of two in-group and two out-group faces. One of each (CS+) was paired with an electrical stimulation (unconditional stimulus, UCS), whereas the other was not coupled with the UCS (CS−). During immediate extinction training, all CS were presented again. On the next day, a retrieval and reinstatement test took place after a stress or a control procedure. Confirming previous research, impaired extinction learning occurred for out-group relative to in-group faces. During the reinstatement test, stress specifically increased responding towards the out-group CS−, thus reducing its safety signaling properties. So, stress seems to reduce the ability to adequately distinguish threat and safety cues after aversive experiences mimicked by reinstatement shocks.

## Introduction

Preparedness theory^1,2^ predicts that fear towards threatening stimuli such as snakes or spiders is learned more readily and cannot be extinguished as easily compared to non-threatening stimuli^3^^; but see^^4^. In particular, this resistance to extinction has also been observed when using out-group faces as conditional stimuli (out-group extinction bias^5–9;^ ^see^^10^ ^for a review^). The idea here is that fear and extinction learning as well as race biases share underlying neural circuits encompassing the amygdala and areas within the prefrontal cortex^11–13^. Such race biases exist on an explicit, but also on an implicit level and can develop into negative judgments and/or aggressive behavior towards out-group members or even manifest xenophobia^14–17^. Thus, investigating factors that promote or reduce such race biases^18^ is crucially important for modern societies in times of major migration flows.

In more detail, Olsson and colleagues^5^ showed that extinction learning was impaired in Caucasian-American participants when Black faces were used as conditioned stimuli (CS), while African-Americans exhibited attenuated extinction learning when confronted with pictures of White faces. Particularly, this out-group extinction bias was found for pictures of men used as CS, but not for pictures of women^9^, comparable to the usage of angry male or female faces as CS^19,20^. This work employing Black and White faces has been replicated in a European sample^8^, in Caucasian Australians with pictures of Chinese faces^7^ and in Caucasian Swedes with Black, but not Middle-Eastern (Moroccan) faces employed as out-group^6^. In order to replicate and expand the current literature, Caucasian-German participants will be confronted with Middle-Eastern faces as out-group CS in the present study.

One factor potentially promoting race biases as mentioned above is stress via its involvement in various learning and memory processes^21–23^. Stress elicits an orchestrated response of the sympathetic nervous system (involving the release of (nor)adrenaline) and the hypothalamus-pituitary-adrenocortical axis (leading to the release of glucocorticoids, mainly cortisol in humans). The stress hormone cortisol impairs memory retrieval^23,24^ targeting especially emotionally arousing material (^meta-analysis:^^21^) and shifts goal-directed and flexible to more habitual behavior^25–27^. During a retrieval or reinstatement test in fear conditioning designs, stress inhibits the retrieval of the extinction memory trace rather than the retrieval of the fear memory trace^28,29;^ ^see^^30,31^ ^for recent reviews^. As such, different observations can be interpreted as impaired extinction retrieval: for example, a reemerging conditioned response (higher responses towards the CS+ compared to the CS−) after successful extinction learning (return of fear) or a specific increase of responding towards the CS− to a similar level as the CS+ (deficit in safety signaling). On the one hand, we hypothesize that stress impairs extinction retrieval. On the other hand, we suppose this stress effect to be especially evident for out-group faces, since stress should promote more habitual behavior^25–27^, in this case more fear towards prepared, threat-associated stimuli as out-group faces ^e.g.^^5^.

## Results

### Fear acquisition and extinction

**Figure 1:**
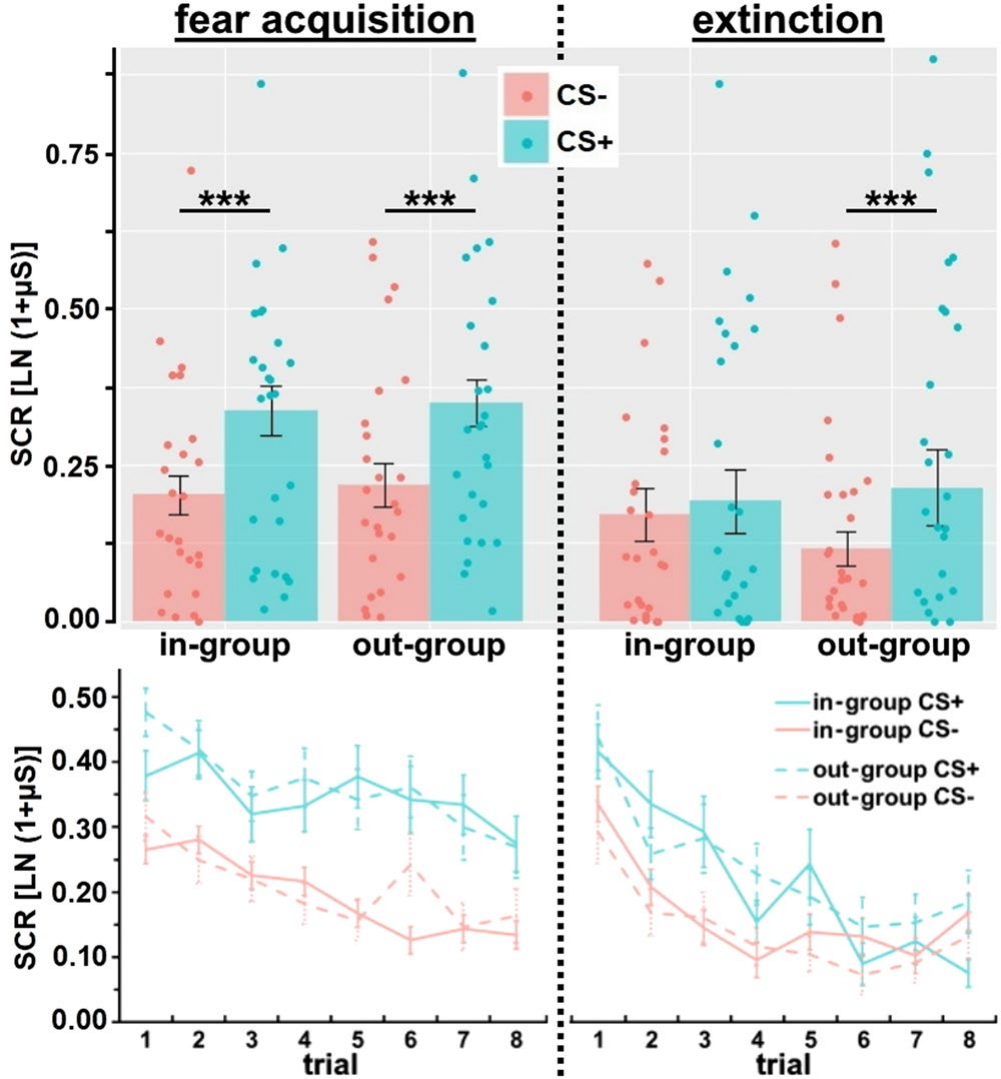
Mean (± s.e.m.) and individual skin conductance responses (SCRs) as well as trial-by-trial plots for in-group and out-group CS+ and CS− during fear acquisition and extinction. Fear acquisition was successful with higher SCRs towards the CS+ than to the CS− for in-group and out-group CS. Successful extinction learning occurred for in-group CS (no significant CS+/CS− difference was observed), but not for out-group CS (CS+/CS− difference was still significant). ***p < 0.005.

Fear acquisition was successful with generally higher skin conductance responses (SCRs) towards the CS+ than to the CS− as indicated by a main effect CS (*F*_(1;49)_ = 35.78, *p* < 0.001, *η*_*p*_^2^ = 0.42) and trial (*F*_(4.8;237.0)_ = 8.78, *p* < 0.001, *η*_*p*_^2^ = 0.15). In-group and out-group faces were not differentially fear conditioned, since no main or interaction effects with face were observed (all *F* < 0.96, all *p* > 0.33). Besides, a significant CS+/CS− difference was observed separately for in-group CS (main effect CS: *F*_(1;49)_ = 30.19, *p* < 0.001, *η*_*p*_^2^ = 0.38) as well as out-group CS (main effect CS: *F*_(1;49)_ = 32.33, *p* < 0.001, *η*_*p*_^2^ = 0.40; cf. Fig. 1). Additionally, we analyzed the end of fear acquisition training to confirm significant differences between CS+ and CS−, which was indeed the case for the mean of the last two trials (*F*_(1;49)_ = 10.97, *p* = 0.002, *η*_*p*_^2^ = 0.18). A separate analysis showed that SCRs were higher towards the CS+ compared to the CS− during the last two trials both for in-group (*F*_(1;49)_ = 16.40, *p* < 0.001, *η*_*p*_^2^ = 0.25) as well as out-group CS (*F*_(1;49)_ = 4.92, *p* = 0.031, *η*_*p*_^2^ = 0.09).

Extinction learning was modulated by face (interaction CS × face: *F*_(1;49)_ = 4.23, *p* = 0.045, *η*_*p*_^2^ = 0.08; main effect CS: *F*_(1;49)_ = 14.98, *p* < 0.001, *η*_*p*_^2^ = 0.23; trial: *F*_(5,3;261.1)_ = 20.75, *p* < 0.001, *η*_*p*_^2^ = 0.30): no significant difference occurred between the in-group CS+ and CS− (*F*_(1;49)_ = 4.66, *p* = 0.072), but the out-group CS+/CS− difference was still significant (*F*_(1;49)_ = 16.76, *p* < 0.001, *η*_*p*_^2^ = 0.26; cf. Fig. 1). Furthermore, we had a closer look at the last two trials of extinction training, which also revealed that extinction learning was modulated by face (interaction CS × face: *F*_(1;49)_ = 6.51, *p* = 0.014, *η*_*p*_^2^ = 0.12): no differences occurred for in-group CS (*F*_(1;49)_ = 1.98, *p* = 0.17), but the CS+/CS− differentiation could still be observed at the end of extinction training for out-group CS (*F*_(1;49)_ = 5.09, *p* = 0.029, *η*_*p*_^2^ = 0.09) indicating successful extinction for in-group, but not for out-group CS.

### Stress responses

**Figure 2:**
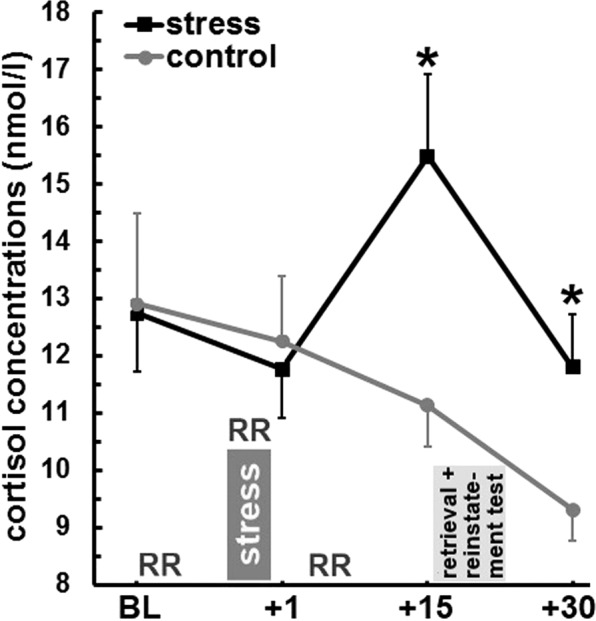
The impact of stress induction on the time course of mean (±s.e.m.) cortisol concentrations. Stress induction via the Socially Evaluated Cold Pressor Test took place after a baseline (BL) measurement of cortisol and blood pressure (RR). During hand immersion into ice-cold (stress group) or warm water (control group) and 5 min afterwards, RR was measured a second and third time (results concerning RR are shown in Table 1). Immediately as well as 15 min and 30 min after the stress/control procedure, further saliva samples were collected. In the stress group, significantly higher cortisol concentrations emerged over the course of the experiment compared to the control group. Particularly, cortisol concentrations were higher before and after the retrieval and reinstatement test took place. *p < 0.05.

The stress induction via the Socially Evaluated Cold Pressor Test (SECPT^32^) was successful as shown by analyses of cortisol concentrations (cf. Fig. 2), blood pressure and ratings (cf. Table 1). Increasing cortisol concentrations over time occurred in the stress compared to the control group (interaction time × stress: *F*_(1.3;65.3)_ = 4.49, *p* = 0.028, *η*_*p*_^2^ = 0.08; main effect time: *F*_(1.3;65.3)_ = 4.94, *p* = 0.021, *η*_*p*_^2^ = 0.09). In particular, the stress group exerted significantly higher cortisol concentrations compared to the control group before (*t*_(45.5)_ = 2.74, *p* = 0.009, *η*_*p*_^2^ = 0.14) and after the retrieval and reinstatement test (*t*_(47.9)_ = 2.29, *p* = 0.026, *η*_*p*_^2^ = 0.10), but neither at baseline (*t*_(49)_ = 0.09, *p* = 0.93) nor immediately after the stress/control procedure (*t*_(49)_ = 0.34, *p* = 0.74).

**Table 1:** Mean (±s.e.m.) systolic and diastolic blood pressure data and ratings by the stress and control group. *P*-values result from independent-sample *t*-tests for the comparison between the stress and control group.

	control	stress	*p*-values
**systolic blood pressure (mmHg)**			
baseline	102.77 ± 2.33	105.47 ± 1.51	0.325
during stress/control procedure	102.90 ± 2.23	118.14 ± 1.83	<0.001
5 min after stress/control procedure	100.63 ± 1.53	103.61 ± 1.53	0.234
**diastolic blood pressure (mmHg)**			
baseline	58.31 ± 1.19	60.99 ± 1.13	0.153
during stress/control procedure	60.04 ± 1.81	72.42 ± 1.08	<0.001
5 min after stress/control procedure	60.23 ± 1.05	62.49 ± 1.19	0.236
**ratings after stress/control condition**			
stressful	1.18 ± 0.09	50.00 ± 4.41	<0.001
painful	0.00 ± 0.00	69.38 ± 4.09	<0.001
unpleasant	2.94 ± 1.87	68.79 ± 3.84	<0.001

The two groups also differed over time in systolic (interaction time × stress: *F*_(2,96)_ = 25.45, *p* < 0.001, *η*_*p*_^2^ = 0.35; main effect time: *F*_(2,96)_ = 38.21, *p* < 0.001, *η*_*p*_^2^ = 0.44; main effect stress: *F*_(1,48)_ = 7.71, *p* = 0.008, *η*_*p*_^2^ = 0.14) and diastolic blood pressure (interaction time × stress: *F*_(2,96)_ = 26.19, *p* < 0.001, *η*_*p*_^2^ = 0.35; main effect time: *F*_(2,96)_ = 37.26, *p* < 0.001, *η*_*p*_^2^ = 0.44; main effect stress: *F*_(1,48)_ = 11.82, *p* = 0.001, *η*_*p*_^2^ = 0.20). Significantly higher systolic and diastolic blood pressure emerged in the stress compared to the control group during hand immersion only (cf. Table 1).

Additionally, the stress group indicated that the SECPT was significantly more stressful (*t*_(35.16)_ = 10.90, *p* < 0.001, *η*_*p*_^2^ = 0.77), painful (*t*_(33)_ = 16.96, *p* < 0.001, *η*_*p*_^2^ = 0.90) and unpleasant (*t*_(45.29)_ = 15.43, *p* < 0.001, *η*_*p*_^2^ = 0.84) compared to the control group.

### Retrieval and reinstatement test

**Figure 3:**
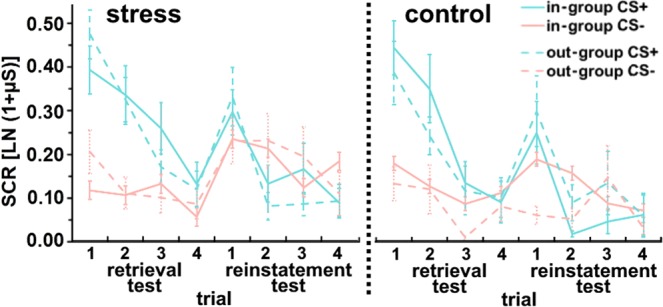
Trial-by-trial plots (depicting mean ± s.e.m) of skin conductance responses (SCRs) for in-group and out-group CS+ and CS− during the four trials of the retrieval test and the four trials of the reinstatement test.

SCRs towards the different CS changed from the end of extinction training to the retrieval test (main effect CS: *F*_(1;49)_ = 49.44, *p < *0.001, *η*_*p*_^2^ = 0.50; main effect time: *F*_(1;49)_ = 19.05, *p < *0.001, *η*_*p*_^2^ = 0.28; interaction CS × time: *F*_(1;49)_ = 39.42, *p < *0.001, *η*_*p*_^2^ = 0.45; interaction CS × in-/out-group face × time: *F*_(1;49)_ = 6.02, *p* = 0.018, *η*_*p*_^2^ = 0.11). Follow up tests revealed higher SCRs towards the CS+ compared to the CS− during the retrieval test (main effect CS: *F*_(1;49)_ = 72.59, *p < *0.001, *η*_*p*_^2^ = 0.59, Fig. 3; for results concerning end of extinction, please see 2.1). Thus, stress did not modulate SCRs during the retrieval test.

Furthermore, a general SCR increase from late retrieval to the early reinstatement test (main effect time: *F*_(1;49)_ = 13.26, *p* = 0.001, *η*_*p*_^2^ = 0.21, Fig. 3) indicated a generalized reinstatement effect. In addition, CS responding was subject to a modulation by in-/out-group face and stress (interaction CS × in-/out-group face × stress: *F*_(1;49)_ = 11.33, *p* = 0.001, *η*_*p*_^2^ = 0.19; main effect CS: *F*_(1;49)_ = 5.72, *p* = 0.001, *η*_*p*_^2^ = 0.11), which was observed across the retrieval and reinstatement test (i.e., independent of the factor time). However, this interaction was observed during the early reinstatement test only (interaction CS × in-/out-group face × stress: *F*_(1;49)_ = 4.98, *p* = 0.030, *η*_*p*_^2^ = 0.09) and not during the last retrieval test (*F*_(1;49)_ = 3.14, *p* = 0.082). For clarity purposes, we focused on SCRs during the early reinstatement test in the following (please note that the same findings were observed when this three-way interaction was further tested using the late retrieval and early reinstatement test in post hoc analyses).

**Figure 4:**
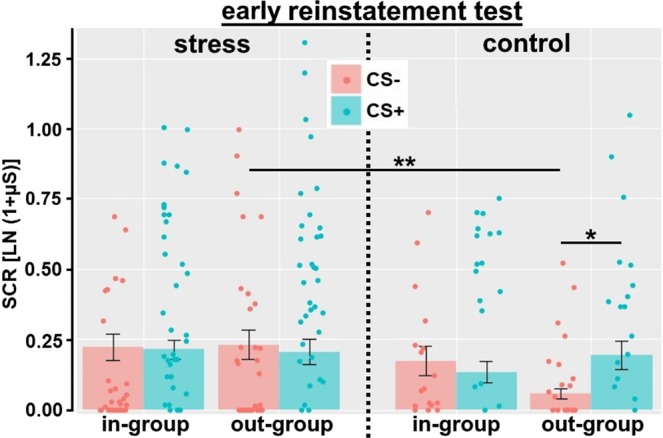
Mean (±s.e.m.) and individual skin conductance responses (SCRs) towards in-group and out-group CS+ and CS− during the early reinstatement test, shown separately for the control and the stress group. Stress increased responding towards the out-group CS−. In addition, a higher CS+/CS− differentiation was found for out-group CS in the control group, but not in the stress group. Thus, stress turned the differential reinstatement effect regarding out-group faces into a generalized reinstatement effect. **p < 0.01; *p < 0.05.

During the early reinstatement test, stress modulated SCRs only for out-group CS (interaction CS × stress: *F*_(1;49)_ = 6.67, *p* = 0.026, *η*_*p*_^2^ = 0.12), but not for in-group CS (*F*_(1;49)_ = 0.18, *p* = 0.68). In more detail, stress increased SCRs towards the out-group CS− (*t*_(39.4)_ = 3.15, *p* = 0.006, *η*_*p*_^2^ = 0.20), but not towards the out-group CS + (*t*_(49)_ = 0.17, *p* = 0.87). In addition, the control group showed a higher CS+/CS− differentiation for out-group CS (*t*_(16)_ = 2.90, *p* = 0.022, *η*_*p*_^2^ = 0.34), which was absent in the stress group (*t*_(33)_ = 0.65, *p* = 0.52; cf. Fig. 4).

## Discussion

The current study replicated and extended previous results concerning the out-group extinction bias. Moreover and importantly, we showed that stress led to higher responding regarding the out-group face, which was never paired with the electrical stimulation (CS−). These results have important implications for understanding implicit and explicit race biases having the potential to develop into xenophobia^14–17^.

Replicating previous research on the out-group extinction bias^5–9^^; see^^10^ ^for a review^, the present study confirmed equivalent fear acquisition for in- and out-group faces, but impaired extinction learning for out-group CS (cf. Fig. 1). The CS+/CS− differentiation was extinguished for in-group CS, but not for out-group CS, which was also evident at the end of extinction training. Thus, extinction learning was only successful for in-group CS and retrieval of out-group CS needs to be interpreted based on their unsuccessful extinction learning. Apart from associative learning processes, other possibilities might account for the present results such as stronger and more persistent learning of novel or unfamiliar stimuli compared to familiar stimuli^33,34^. This alternative explanation would assume that extinction learning of out-group faces is not impaired, but actually intact due to superior conditioning^35^. Furthermore, pre-existing expectancy biases should be mentioned assuming that people more likely couple threat-associated, prepared stimuli with an aversive event compared to non-threatening stimuli^36,37^.

Stress targeted the extinction memory trace and reduced its inhibitory properties over the fear memory trace, eventually leading to higher fear-related responding towards out-group faces, which was evident in the stress group during the reinstatement test. Stress specifically enhanced responding towards the out-group CS− (cf. Fig. 4), thus reducing its safety signaling properties. These findings are in line with previous research on stress effects on declarative memory^21,23,24^ and fear conditioning^28,29^^; see^^30,31^ ^for recent reviews^. Critically, the ability to distinguish threat and safety cues, even after aversive experiences, is essential as protecting factor against the development of pathological anxiety^38–40^. Stress turned the differential reinstatement effect (higher SCRs towards the CS+ compared to the CS−) observed for out-group faces in the control group (which can be interpreted as a continuation of fear responding or fear retrieval in the face of unsuccessful extinction learning for out-group CS; cf. Fig. 1) into a generalized reinstatement effect (increased responding towards the CS− to a similar level as the CS+; cf. Fig. 4). Similar findings to this stress effect regarding reinstatement have been reported for recently experienced adversity^41^ and state anxiety^42^. Thus, stress and anxiety related states and traits could promote generalization processes from danger to safety cues and/or inhibit fear in the presence of safety cues. On the neural level, it has already been shown that cortisol administration leads to increased amygdala signaling after reinstatement^28^, which might be even more pronounced for out-group faces. In light of previous work^6^ showing the out-group extinction bias in Caucasian Swedes with Black, but not Moroccan faces (also used in the present study), it remains to be shown how the present results extend to a different contextual and cultural embedding.

Additionally, our findings fit well to the idea that stress shifts goal-directed, cognitively demanding and flexible behavior promoted by the hippocampus and prefrontal areas to more habitual behavior driven by the dorsal striatum^25–27^, in our case more fear towards prepared, threat-associated stimuli such as out-group faces. For example, a reduced memory flexibility after stress would explain the absent differentiation between out-group CS+ and CS− during the reinstatement test. Additionally, we assume persisting learned threat stereotypes and/or implicit negative associations towards out-group faces, existing even after extinction training. These stereotypes and associations might particularly emerge after stressful situations, which in turn lead to more habitual and rigid behavior.

Since out-group faces also impair flexibility of learning experiences as evident in fear reversal learning^43^, one step forward would comprise ideas how to reduce the out-group extinction bias to allow for flexible updating of experiences. For example, future studies could further characterize beneficial effects of positive intergroup contact^5,44–46^, storytelling/verbal instructions concerning out-group members^7,47^ or multiple out-group stimuli and/or contexts during extinction training ^cf.^^48^ on the out-group extinction bias. In the presence of current major migration flows, the contribution of such strategies to possibly attenuate stress effects promoting deficient out-group related processing of safety signals should be evaluated.

## Methods

### Participants

Fifty-seven healthy women (mostly students except two) were recruited at the Ruhr University Bochum via email announcements or flyers. We decided to include only women in this experiment, since race biases differ between men and women: while men’s behavior is more driven by aggression and social dominance towards male out-group stimuli^49^ (cf. Stimulus material and presentation), women’s behavior is more based on fear. In a standardized telephone interview, the following exclusion criteria were checked: report of chronic or acute illnesses, regular intake of medicine (including hormonal contraceptives), current medical or psychotherapeutic treatment, drug use including smoking, body mass index (BMI) < 18 kg/m² or >27 kg/m², age < 18 years or >35 years, no German descent until grandparents, working in shift work and vaccination during the last two weeks prior to the experiment, traveling to a country with a time difference >5 h or blood donation in the last month.

Participants were instructed not to drink alcohol the day before and between the two testing sessions. In addition, they should refrain from eating or drinking anything but water and not to do any exhausting physical exercise 90 min before the start of the sessions. At the end of the second testing session, participants received either partial course credits or 25€ as compensation. All procedures were in accordance with the Declaration of Helsinki and approved by the ethics committee of the Faculty of Psychology at the Ruhr University Bochum.

Six participants were excluded from all analyses due to termination of the experiment because of circulation problems (*n* = 2), not showing up for the second testing session (*n* = 2) or technical failure of the skin conductance recording system (*n* = 2). Thus, the final sample consisted of 51 women (age: 18–31; mean age ± s.d.: 24.1 ± 3.2 years) with 17 women in the control group and 34 women in the stress group (cf. Stress and control procedure). The sample size of 17 was derived from G*Power 3.1^50^ for a repeated-measures analysis of variance (ANOVA), assuming an medium size effect of stress hormones on memory retrieval (*d* = −0.49) as reported in a meta-analysis^21^. In order to detect a significant CS × in-/out-group face × stress interaction during the retrieval and reinstatement test (cf. Skin conductance responses (SCRs): measurements and analyses) with a 95% power and an α-level of 0.05, a total sample size of 34 participants was required, thus, 17 women in the control group exactly matches this power analysis. We decided to include twice as much participants in the stress relative to the control group ^cf.^^51^. to have enough power to correlate cortisol increases with conditioned responding (accounting for individual differences after exposure to stress) during the retrieval and reinstatement test (which did not result in any significant correlation, data not shown).

### Stimulus material and presentation

Four pictures of neutral male faces were taken from the Radboud Faces Database^52^ and used as conditional stimuli (CS). They were presented for 6 s on a 19-inch computer screen positioned approximately 50 cm in front of the participants using Presentation® software (version 18.0, Neurobehavioral Systems, Inc., Berkeley, CA, www.neurobs.com). Two male faces belonged to the Caucasian race representing the in-group (picture codes: Rafd090_23, Rafd090_33), while the other two faces were Moroccan faces representing the out-group for the participants (picture codes: Rafd090_69, Rafd090_70). We used male faces, since the out-group extinction bias was found for pictures of men used as CS, but not for pictures of women^9^. More generally, race biases also more likely occur for male target faces^49^. One face of each group (CS+, in-group vs. out-group) was coupled with the unconditional stimulus (UCS), while the other face was never paired (CS−). Allocation of the in-group and out-group CS+ and CS− was counterbalanced and matched between the control and stress group. A black screen with a white fixation cross (located at the level of the eyes of the male faces) was shown during the 15 s inter-trial intervals between the end of a CS presentation and the start of the next CS presentation.

A transcutaneous electrical stimulation was used as UCS occurring 5.9 s after onset of the in-group and out-group CS+. A constant voltage stimulator (STM200; BIOPAC Systems, Inc.) delivered 100 ms electrical stimulation via two Ag/AgCl electrodes filled with isotonic electrolyte medium (Synapse Conductive Electrode Cream, Kustomer Kinetics, Inc., Arcadia, CA) fixed to the middle of the left shin. Using a gradually increasing rating procedure, participants chose the intensity of the electrical stimulation individually to be “unpleasant but not painful”. Stimulation electrodes remained attached during all phases of the fear conditioning procedure, but only delivered electrical stimulation during fear acquisition training (in total ten times signalled by the CS+) and during reinstatement (in total four times unsignalled). Reinstatement took place after the retrieval and before the reinstatement test without any interruption. During reinstatement, a gray screen was presented for 20 s and UCS applications were separated by 5 s intervals starting with 2 s after onset of the gray screen.

Pseudo-randomized stimulus orders were realized using eight blocks each for fear acquisition and extinction training and four blocks each for the retrieval and reinstatement test. Each block contained one presentation of each of the four CS (randomly distributed), only reinforced in-group and out-group CS+ presentations were used during the first and last block during acquisition training and generally, no more than two consecutive presentations of a CS+ or CS− were allowed. Stimulus presentation orders and CS allocation were matched between the stress and the control group.

### Stress and control procedure

Participants were randomly assigned to either the Socially Evaluated Cold Pressor Test (SECPT^32^; *n* = 34) or a non-stressful control procedure (^cf.^^32^; *n* = 17). In the SECPT, participants should immerse their right hand and wrist in a basin filled with ice-cold water (0–2 °C) for a maximum time of 3 min, while being video recorded and monitored by an unknown, reserved acting male experimenter. In the control procedure, participants should immerse their right hand into warm water (36–37 °C) for 3 min without videotaping and monitoring.

### General procedure

Individual testing sessions took place at the same time (±30 min) between 9 a.m. and 1p.m. of two consecutive days to control for the circadian cortisol rhythm varying over the course of the day. During an initial resting phase of 20 min, participants were informed about the course of the experiment (application of electrical stimulations, SCR measurement, stress induction, saliva sampling) and given the possibility to ask questions. After that, they provided written informed consent, and filled out questionnaires on demographic variables. Instructions of the experiment comprised the information that throughout the whole experiment participants may or may not receive an electrical stimulation after presentation of a particular face. If a face was secure, this face would always be secure; if a face was followed by an electrical stimulation, this might or might not happen again after presentation of this particular face.

During fear acquisition training, the in-group and out-group CS+ were followed by an aversive electrical stimulation in five out of eight trials (62.5% reinforcement rate), whereas the in-group and out-group CS− were presented eight times alone and intermixed with the CS+ presentations. During the subsequent extinction training, all four CS were shown eight times without any pairing with the UCS.

About 24 h (+/−30 min) later, participants were exposed either to the stress or control procedure. Approximately 20 min after stress offset, the retrieval test started with four presentations of all four CS again not coupled with the UCS. Without any interruption, reinstatement with four UCS applications and the reinstatement test with four presentations of all four CS followed.

### Stress responses: measurements and analyses

Saliva samples were collected using Salivette sampling devices (Sarstedt, Nümbrecht, Germany) 5 min before the start of the stress or control procedure (baseline), +1 min, +15 min and +30 min after the end of the procedure. All saliva samples were stored at −20 °C until free cortisol concentrations were analyzed on a Synergy2 plate reader (Biotek, USA) using a commercial enzyme-linked immunosorbent assay (ELISA; Demeditec, Kiel, Germany) according to the manufacturer’s instructions. Inter- and intra-assay variations were below 8% and 6% respectively.

Systolic and diastolic blood pressure were measured using a Dinamap vital signs monitor (Criticon, Tampa, FL; cuff placed on the left upper arm) before, during, and after hand immersion into ice-cold or warm water. Due to technical malfunctioning, data on blood pressure for one participant are missing. Directly after offset of the stress or control procedure, participants rated how stressful, painful, and unpleasant the respective procedure was on a scale from 0 (“not at all”) to 100 (“very much”; ratings adopted from^32^).

Repeated-measures ANOVA were conducted separately for cortisol, systolic and diastolic blood pressure including the repeated measurement factor time (four measurements for cortisol, three for systolic and diastolic blood pressure) as well as the between-subjects factor stress (stress vs. control). Two-tailed two-sample t-tests served to test differences between the stress and the control group in perceived stressfulness, painfulness, and unpleasantness during the experimental condition.

### Skin conductance responses (SCRs): measurements and analyses

SCRs were sampled at a sampling rate of 1000 Hz with a commercial SCR coupler and amplifying system (MP150 + GSR100C, BIOPAC Systems, Inc; software: Acqknowledge 4.2). Ag/AgCl electrodes filled with isotonic electrolyte medium (Synapse Conductive Electrode Cream) were attached to the hypothenar on the non-dominant hand. A high pass filter with a cut-off frequency of 0.05 Hz was applied to raw SCR data. Conditioned SCRs were defined as the trough-to-peak amplitude difference of the largest deflection (in µS; minimum amplitude threshold: 0.01µS) starting within a window of 1–6.5 s after CS onset as before^53^. The natural logarithm was applied to SCR data in order to attain a normal distribution.

Statistical comparisons of SCRs were conducted separately for each phase (fear acquisition training, extinction training, retrieval test and reinstatement test) via repeated-measures ANOVA. For fear acquisition and extinction training, the between-subject factor stress was entered as well as the within-subject factors CS (CS+ vs. CS−), in-/out-group face and trial (comprising eight trials for each CS). Additionally, the end of fear acquisition and extinction training was analyzed by comparing the mean of the last two trials in a 2 (CS) × 2 (in-/out-group face) ANOVA. For the retrieval test, mean SCRs for the last two extinction trials were compared with the first two retrieval test trials using a CS × in-/out-group face × time × stress repeated-measures ANOVA. For the reinstatement test, mean SCRs for the last two retrieval test trials were compared with the first two reinstatement test trials ^cf.^^54^ using a CS × in-/out-group face × time × stress repeated-measures ANOVA. Please note that in addition to trial-by-trial analyses and plots we consistently used the mean of two trials per CS for all analyses, which reflects the beginning or end of the respective experimental phase. Thus, our reported comparisons always include the same number of included trials for consistency sake. Since fear conditioning paradigms differ enormously in their designs (including number of CS and trials), there is no common practice regarding how many trials should be included in such analyses ^cf.^^55^.

All statistical analyses were performed in IBM SPSS Statistics for Windows (Version 21.0. Armonk, NY: IBM Corp.) with the statistical significance level set to *α* = 0.05. Greenhouse-Geisser corrected *p*-values were used if assumptions of sphericity were violated. Significant results for ANOVA comparisons were analyzed in adequate post hoc tests with Bonferroni-corrected *p*-values according to the number of comparisons in order to minimize Type I errors. We reported only significant main and interaction effects for the overarching ANOVA.

## Data Availability

The data sets analyzed during the current study are available at the Open Science Framework (OSF) under https://osf.io/9ubse/.

## References

[1] Seligman MEP (1970). On the generality of the laws of learning. Psychol. Rev..

[2] Seligman MEP (1971). Phobias and preparedness. Behav. Ther..

[3] Öhman A, Mineka S (2001). Fears, phobias, and preparedness: toward an evolved module of fear and fear learning. Psychol. Rev..

[4] Åhs F (2018). Biological preparedness and resistance to extinction of skin conductance responses conditioned to fear relevant animal pictures: a systematic review. Neurosci. Biobehav. Rev..

[5] Olsson A, Ebert JP, Banaji MR, Phelps EA (2005). The role of social groups in the persistence of learned fear. Sci..

[6] Golkar A, Bjornstjerna M, Olsson A (2015). Learned fear to social out-group members are determined by ethnicity and prior exposure. Front. Psychol..

[7] Mallan MK, Sax J, Lipp OV (2009). Verbal instruction abolishes fear conditioned to racial out-group faces. J. Exp. Soc. Psychol..

[8] Molapour T, Golkar A, Navarrete CD, Haaker J, Olsson A (2015). Neural correlates of biased social fear learning and interaction in an intergroup context. Neuroimage.

[9] Navarrete CD (2009). Fear extinction to an out-group face: the role of target gender. Psychol. Sci..

[10] O’Donnell, A. W., Neumann, D. L., Duffy, A. L. & Paolini, S. Learning to fear outgroups: an associative learning explanation for the development and reduction of intergroup anxiety. *Soc Personal Psychol Compass* **13**; 10.1111/spc3.12442 (2019).

[11] Phelps EA (2000). Performance on indirect measures of race evaluation predicts amygdala activation. J. Cogn. Neurosci..

[12] Amodio DM (2014). The neuroscience of prejudice and stereotyping. Nat. Rev. Neurosci..

[13] Kubota JT, Banaji MR, Phelps EA (2012). The neuroscience of race. Nat. Neurosci..

[14] Payne BK (2001). Prejudice and perception: the role of automatic and controlled processes in misperceiving a weapon. J. Pers. Soc. Psychol..

[15] Devine PG (2001). Implicit prejudice and stereotyping: how automatic are they? Introduction to the special section. J. Pers. Soc. Psychol..

[16] Sagar HA, Schofield JW (1980). Racial and behavioral cues in black and white children’s perceptions of ambiguously aggressive acts. J. Pers. Soc. Psychol..

[17] Faulkner J, Schaller M, Park JH, Duncan LA (2004). Evolved disease-avoidance mechanisms and contemporary xenophobic attitudes. Group. Process. Intergroup Relat..

[18] Kelly D, Faucher L, Machery E (2010). Getting rid of racism: assessing three proposals in light of psychological evidence. J. Soc. Philos..

[19] Mazurski EJ, Bond NW, Siddle DA, Lovibond PF (1996). Conditioning with facial expressions of emotion: effects of CS sex and age. Psychophysiol..

[20] Öhman A, Dimberg U (1978). Facial expressions as conditioned stimuli for electrodermal responses: a case of “preparedness”. J. Pers. Soc. Psychol..

[21] Shields GS, Sazma MA, McCullough AM, Yonelinas AP (2017). The effects of acute stress on episodic memory: a meta-analysis and integrative review. Psychol. Bull..

[22] Schwabe L, Joëls M, Roozendaal B, Wolf OT, Oitzl MS (2012). Stress effects on memory: an update and integration. Neurosci. Biobehav. Rev..

[23] Wolf OT (2009). Stress and memory in humans: twelve years of progress?. Brain Res..

[24] Wolf OT (2017). Stress and memory retrieval: mechanisms and consequences. Curr. Opin. Behav. Sci..

[25] Schwabe L, Wolf OT (2013). Stress and multiple memory systems: from ‘thinking’ to ‘doing’. Trends Cogn. Sci..

[26] Quaedflieg CWEM, Schwabe L (2018). Memory dynamics under stress. Mem..

[27] Goldfarb EV, Phelps EA (2017). Stress and the trade-off between hippocampal and striatal memory. Curr. Opin. Behav. Sci..

[28] Kinner VL, Wolf OT, Merz CJ (2018). Cortisol increases the return of fear by strengthening amygdala signaling in men. Psychoneuroendocrinology.

[29] Raio CM, Brignoni-Perez E, Goldman R, Phelps EA (2014). Acute stress impairs the retrieval of extinction memory in humans. Neurobiol. Learn. Mem..

[30] Meir Drexler S, Merz CJ, Jentsch VL, Wolf OT (2019). How stress and glucocorticoids timing-dependently affect extinction and relapse. Neurosci. Biobehav. Rev..

[31] Merz CJ, Wolf OT (2017). Sex differences in stress effects on emotional learning. J. Neurosci. Res..

[32] Schwabe L, Haddad L, Schächinger H (2008). HPA axis activation by a socially evaluated cold-pressor test. Psychoneuroendocrinology.

[33] Dunsmoor JE, Niv Y, Daw N, Phelps EA (2015). Rethinking extinction. Neuron.

[34] Courville AC, Daw ND, Touretzky DS (2006). Bayesian theories of conditioning in a changing world. Trends Cogn. Sci..

[35] Maia TV (2009). Fear conditioning and social groups: statistics, not genetics. Cogn. Sci..

[36] Davey GCL (1992). An expectancy model of laboratory preparedness effects. J. Exp. Psychol. Gen..

[37] Tomarken AJ, Mineka S, Cook M (1989). Fear-relevant selective associations and covariation bias. J. Abnorm. Psychol..

[38] Lissek S (2005). Classical fear conditioning in the anxiety disorders: a meta-analysis. Behav. Res. Ther..

[39] Duits P (2015). Updated meta-analysis of classical fear conditioning in the anxiety disorders. Depress. Anxiety.

[40] Craske MG (2012). Elevated responding to safe conditions as a specific risk factor for anxiety versus depressive disorders: evidence from a longitudinal investigation. J. Abnorm. Psychol..

[41] Scharfenort R, Menz M, Lonsdorf TB (2016). Adversity-induced relapse of fear: neural mechanisms and implications for relapse prevention from a study on experimentally induced return-of-fear following fear conditioning and extinction. Transl. psychiatry.

[42] Kuhn M, Mertens G, Lonsdorf TB (2016). State anxiety modulates the return of fear. Int. J. Psychophysiol..

[43] Dunsmoor JE, Kubota JT, Li J, Coelho CAO, Phelps EA (2016). Racial stereotypes impair flexibility of emotional learning. Soc. Cogn. Affect. Neurosci..

[44] Allport, G. W. *The nature of prejudice* (Addison Wesley, Reading, MA, 1954).

[45] Pettigrew TF, Tropp LR (2006). A meta-analytic test of intergroup contact theory. J. Pers. Soc. Psychol..

[46] Paolini S, Harris NC, Griffin AS (2016). Learning anxiety in interactions with the outgroup: towards a learning model of anxiety and stress in intergroup contact. Group. Process. Intergroup Relat..

[47] Husnu S, Mertan B, Cicek O (2018). Reducing Turkish Cypriot children’s prejudice toward Greek Cypriots: vicarious and extended intergroup contact through storytelling. Group. Process. Intergroup Relat..

[48] Shiban Y, Schelhorn I, Pauli P, Mühlberger A (2015). Effect of combined multiple contexts and multiple stimuli exposure in spider phobia: a randomized clinical trial in virtual reality. Behav. Res. Ther..

[49] Navarrete CD, McDonald MM, Molina LE, Sidanius J (2010). Prejudice at the nexus of race and gender: an outgroup male target hypothesis. J. Pers. Soc. Psychol..

[50] Faul F, Erdfelder E, Lang A-G, Buchner A (2007). G*Power 3: a flexible statistical power analysis program for the social, behavioral, and biomedical sciences. Behav. Res. Methods.

[51] Larra MF (2014). Heart rate response to post-learning stress predicts memory consolidation. Neurobiol. Learn. Mem..

[52] Langner O (2010). Presentation and validation of the Radboud Faces Database. Cogn. Emot..

[53] Merz CJ, Hamacher-Dang TC, Wolf OT (2014). Exposure to stress attenuates fear retrieval in healthy men. Psychoneuroendocrinology.

[54] Haaker J, Lonsdorf TB, Thanellou A, Kalisch R (2013). Multimodal assessment of long-term memory recall and reinstatement in a combined cue and context fear conditioning and extinction paradigm in humans. PLoS One.

[55] Lonsdorf TB (2017). Don’t fear ‘fear conditioning’: methodological considerations for the design and analysis of studies on human fear acquisition, extinction, and return of fear. Neurosci. Biobehav. Rev..

